# Chemical Constitution and Antimicrobial Activity of Kombucha Fermented Beverage

**DOI:** 10.3390/molecules26165026

**Published:** 2021-08-19

**Authors:** Abdul-Raouf Al-Mohammadi, Ahmed A. Ismaiel, Rehab A. Ibrahim, Ahmed H. Moustafa, Azza Abou Zeid, Gamal Enan

**Affiliations:** 1 Department of Sciences, King Khalid Military Academy, Riyadh 11459, Saudi Arabia; almohammadi26@hotmail.com; 2 Department of Botany and Microbiology, Faculty of Science, Zagazig University, Zagazig 44519, Egypt; ahmedismaiel@zu.edu.eg (A.A.I.); rehabatef@yahoo.com (R.A.I.); azza.abozeid@yahoo.com (A.A.Z.); 3 Department of Chemistry, Faculty of Science, Zagazig University, Zagazig 44519, Egypt; ah_hu_mostafa@yahoo.com

**Keywords:** kombucha tea, kombucha fermented beverage (KFB), GC-MS analysis, pathogenic bacteria and fungi, antimicrobial activity, Egyptian fruit juices

## Abstract

Kombucha is a traditional beverage of sweetened black tea fermented with a symbiotic association of acetic acid bacteria and yeasts. In this study, kombucha fermented beverage (KFB) appeared to include nine chemical groups (alcohols, acids, lactones, condensed heterocyclic compounds, antibiotics, esters, aldehydes, fatty acids, and alkaloids) of many bioactive metabolites, as elucidated by gas chromatography–mass spectrometry (GC-MS) and IR spectra. The fermented metabolic components of KFB seem collectively to act in a synergistic action giving rise to the antimicrobial activity. Four types of kombucha preparations (fermented, neutralized, heat-treated and unfermented) were demonstrated with respect to their antimicrobial activity against some pathogenic bacterial and fungal strains using agar well diffusion assay. KFB exerted the strongest antimicrobial activities when compared with neutralized and heat-treated kombucha beverages (NKB and HKB). *Staphylococcus aureus* ATCC6538 (*S. aureus*) and *Escherichia coli* ATCC11229 (*E. coli*) were the organisms most susceptible to the antimicrobial activity of kombucha beverage preparations. Finally, the KFB preparation showed remarkable inhibitory activity against *S. aureus* and *E. coli* bacteria in a brain heart infusion broth and in some Egyptian fruit juices (apple, guava, strawberry, and tomato). These data reveal that kombucha is not only a prophylactic agent, but also appears to be promising as a safe alternative biopreservative, offering protection against pathogenic bacteria and fungi.

## 1. Introduction

Probiotics are live microorganisms that when administered in adequate amounts, improve many nutritional and digestive functions [1,2]. After a long history of safe use of probiotics in fermented food products and an increased recognition of their beneficial human health effects, in addition to their significant potential use as therapeutic options for a variety of diseases, the food industry has become increasingly interested in these types of microorganisms [3,4,5,6,7,8,9,10]. The mechanisms underlying the beneficial effects of probiotics are likely to be multifactorial. These mechanisms include production of various antimicrobial agents, modification of the gut microbiota, competitive adherence to the mucosa and epithelium, strengthening of the gut epithelial barrier and modulation of the immune system [1]. The most widely used probiotics to date are several genera of both lactic and acetic acid bacteria as well as yeasts; they are applied in many functional foods and dietary supplements [1]. Furthermore, probiotics have been reported to display a protective role by direct competing with intestinal pathogens through the release of antibacterial substances such as bacteriocins that could kill the multidrug resistant bacteria [4,11,12,13] or metabolites such as acetic acid and lactic acid [1,10]. Probiotic yeasts such as *Saccharomyces cerevisiae* and *S. boulardii* have also been demonstrated to confer health benefits [14,15]. Thus, there is an urgent need to continue research on other probiotic sources with unique prophylactic and antimicrobial characteristics.

Kombucha is a slightly sweet and acidic beverage that is generally prepared by fermenting sweetened black tea with the tea fungus, a symbiotic culture of bacteria and yeast (SCOBY). This symbiotic consortium includes mainly acetic acid bacteria and different yeasts [16]. Kombucha colony/mat represents a symbiotic relationship of yeasts and bacteria, and their composition is highly variable. As reviewed by Jayabalan et al. [16], the most abundant bacteria in this culture belong to the genera *Acetobacter* and *Gluconobacter*. The main bacterium is *Acetobacter xylinum*. In addition to acetic acid bacteria, a broad spectrum of yeast species belonging to *Saccharomyces, Saccharomycodes, Schizosaccharomyces, Zygosaccharomyces*, *Brettanomyces*, *Candida*, *Torulospora*, *Kloeckera*, *Pichia*, *Mycotorula*, and *Mycoderma* are found in kombucha. The basis of symbiotic relationship coincides in the mechanism by which yeasts convert sucrose into glucose and fructose by invertase and produce ethanol via metabolic pathways. Acetic acid bacteria convert glucose to gluconic acid and ethanol to produce acetic acid [16]. Fermentation of kombucha results in the formation of two portions: a floating cellulosic pellicle layer and the sour liquid broth (fermented broth) [17].

Kombucha displayed potential prophylactic activities including weight loss, treatment of metabolic diseases, arthritis, indigestion, cancer and acquired immunodeficiency syndrome (AIDS) [18,19,20]. Regular ingestion of kombucha beverage showed a significant role in weight gain inhibition and life elongation [21]. The prophylactic and antioxidant activities of kombucha are attributed to a variety of active components produced during fermentation, including organic acids (mainly acetic, gluconic, glucuronic acid), sugars (sucrose, glucose, and fructose), water-soluble vitamins (B1, B6, B12 and C), amino acids, biogenic amines, purines, pigments, lipids, proteins, hydrolytic enzymes, ethanol, carbon dioxide, polyphenols, minerals, anions D-saccharic acid-1,4-lactone, and metabolic products of yeasts and bacteria [22].

Kombucha has been reported to exert antimicrobial activity against *Candida krusei* CCM 8271, *C. glabrata* CCM 8270, *C. albicans* CCM 8186, *C. tropicalis* CCM 8223, *Haemophilus influenzae* CCM 4454 and *Escherichia coli* CCM 3954 [9], *Staphylococcus epidermidis* CIP 106510, *S. aureus* ATCC 25923, *Micrococcus luteus* NCIMB 8166, *Salmonella typhimurium* LT2, *E. coli* ATCC 35218, *Listeria monocytogenes* ATCC 19115, *Pseudomonas aeruginosa* ATCC 27853 [23]. Kombucha has been found to contain many metabolites with antimicrobial activity such as organic acids, in particular acetic acid and catechins, in addition to antibiotic substances which were found to inhibit Gram-positive and Gram-negative microorganisms [24].

The consumption of non-dairy nutraceuticals, increased people with lactose intolerance, and the tendency of individuals to veganism are strong justifications to find new, safe, non-dairy probiotic products with prophylactic activities [25,26]. Kombucha and fermented herb drinks may be alternatives for functional dairy products for individuals with lactose intolerance [27]. In this study, we demonstrated kombucha’s antimicrobial activity in vitro and in fruit juices. The feasibility of application of kombucha as an additive in fruit juices in order to protect unrefrigerated juices from bacterial and fungal contamination was investigated. Furthermore, the kombucha’s bioactive compounds by means of available instrumental analysis were identified.

## 2. Results

Kombucha fermented beverage (KFB) was prepared under our experimental conditions using black tea (1.2%) sweetened with sucrose (10%) at an initial pH of 5.0 and inoculated with 3% (*w*/*v*) freshly grown tea fungus followed by incubation at 30 °C for about 14 days. The final pH was found to be decreased to 3.0.

**Figure 1:**
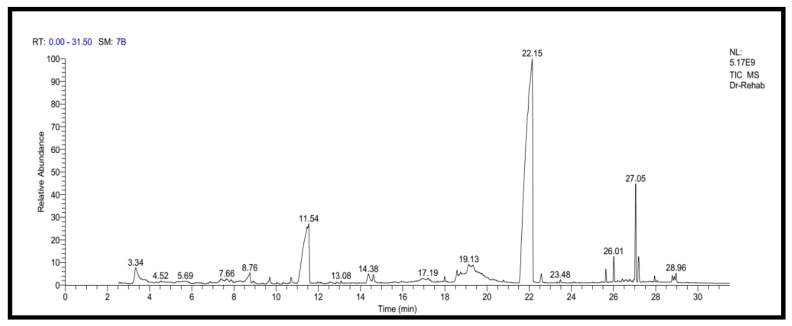
GC-MS analysis of KFB.

**Figure 2:**
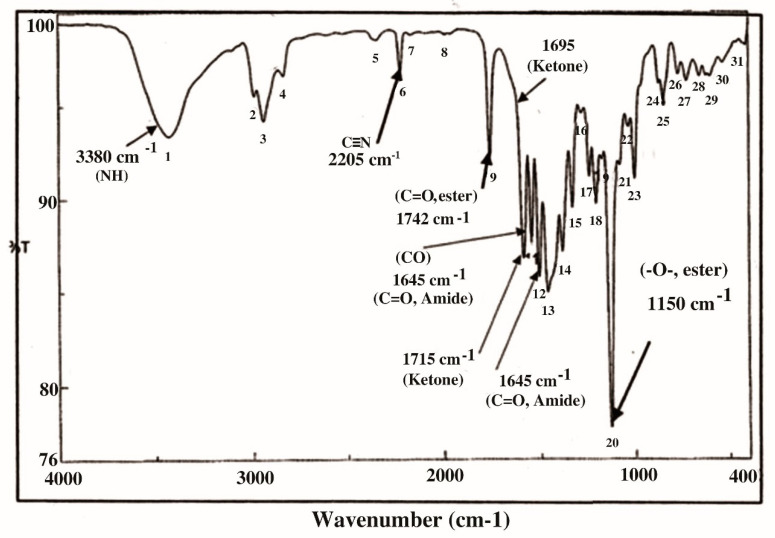
IR spectrum in KBr (discs) for the extraction of KFB.

In the current study, KFB was subjected to GC-MS analysis to detect its bioactive compounds. The results obtained (Table 1 and Figure 1) represent the names and classes, in addition to molecular formula and molecular weight, of the nine chemical categories produced. The main compounds in the KFB preparation were heterocylic alcohols: 2-hydroxy methyl furan, 2-(4-hydroxyphenyl) ethanol; heterocyclic acids: 1,2,3,4-tetrahydro-2,4 dioxo-5-carboxymethyluracil, hexadecanoic acid, ethyl-2-[2,2-dimethylcyclopropanecarboxylate (cyclopropane carboxylic acid); lactone: 3,5-dihydroxy-6-methyl-2,3-diydroxy-4*H*-pyran-4-one, 4-hexyl-2,5-dihydro-2,5-dioxo-3-furan acetic acid, 3-methoxy-2,4,6-trimethylcyclohex-2-enone, 3,7-dimethyl-1-[2-(vinyloxy) ethyl-3,7-dihydro-1*H*-purine-2,6-dione, S-[(2E)-1,3-diphenyl-2-butenyl] dimethylthio carbamate; condensed heterocyclic cpd: 2,3-dihydrobenzofuran, 7,7-dimethyl-1-isobutylhexahydro-2-benzofuran-3a (3*H*)-ol; antibiotics: cypermethrin, cyhalothrin; heterocyclic esters: 5-acetoxymethyl-2-furaldehyde; heterocyclic aldehydes: 5-hydroxymethyl furfural; unsat. fatty acids: 2-hexadecenoic acid; alkaloides: 1,3,7-trimethyl-3,7-dihydro-1 *H*-purine-2,6-dione. In addition, the IR spectrum (Figure 2) showed the presence of bands at 3380 cm^−1^ for CH, 2205 cm^−1^ for C≡N, 1742 cm^−1^ C=O for ester, 1715 cm^−1^ C=O for ketone, 1695 cm^−1^ C=O for aldehyde conjugated with double bond, 1645 cm^−1^ C=O for amide and at 1615 cm^−1^ for C=N. In addition, a band at 1150 cm^−1^ was characterized for the -O-, ether.

To check the bacterial status of kombucha, it was analyzed microbiologically on suitable media as described in the Materials and Methods. The microbial isolates were automatically identified by Viteck-2; the bacterial strains were found to belong *Acetobacter xylinum, A. pasteurians, A. aceti* (acetic acid bacteria), *Lactobacillus fermentum* and *L. acidophilus* (lactic acid bacteria). Additionally, the following yeasts were identified by API-yeasts kits: *Saccharomyces cerevisiae* and *Schizosaccharomyces pombe*. All the microbial cultures were studied with respect to their cell morphology and Gram staining. The microbial isolates were classified into two Gram-positive rods (lactic acid bacteria) and three Gram-negative rods (acetic acid bacteria). The vegetative yeast cultures were Gram-positive oval-shaped cells.

The antibacterial activities of the studied kombucha beverages, viz., KFB, NKB, HKB and UKB, against the pathogenic microorganisms tested are presented in Table 2. The results showed that KFB, NKB, and HKB have antimicrobial potency against all tested bacteria and fungi with varied activity. The three preparations can be arranged in the following descending order according to their antimicrobial potential: KFB ˃ HKB ˃ NKB. The unfermented UKB preparation showed no antimicrobial activity. Significant inhibition zone diameters of all the tested bacterial and fungal strains were obtained with the KFB preparation, compared with other kombucha preparations. Among the bacterial strains tested, *S. aureus* and *E. coli* were the most sensitive organisms, recording 19- and 18-mm inhibition zone diameters, respectively, upon employing KFB preparation and 12-mm inhibition zone diameter of both strains upon employing HKB preparation. The two preparations showed a good antifungal activity against *A. flavus* and *A. niger* with inhibition zones of about 12 and 9.5 mm, respectively, in the case of the KFB preparation and 5.0 and 4.6 mm, respectively, in the case of the HBK preparation.

**Figure 3:**
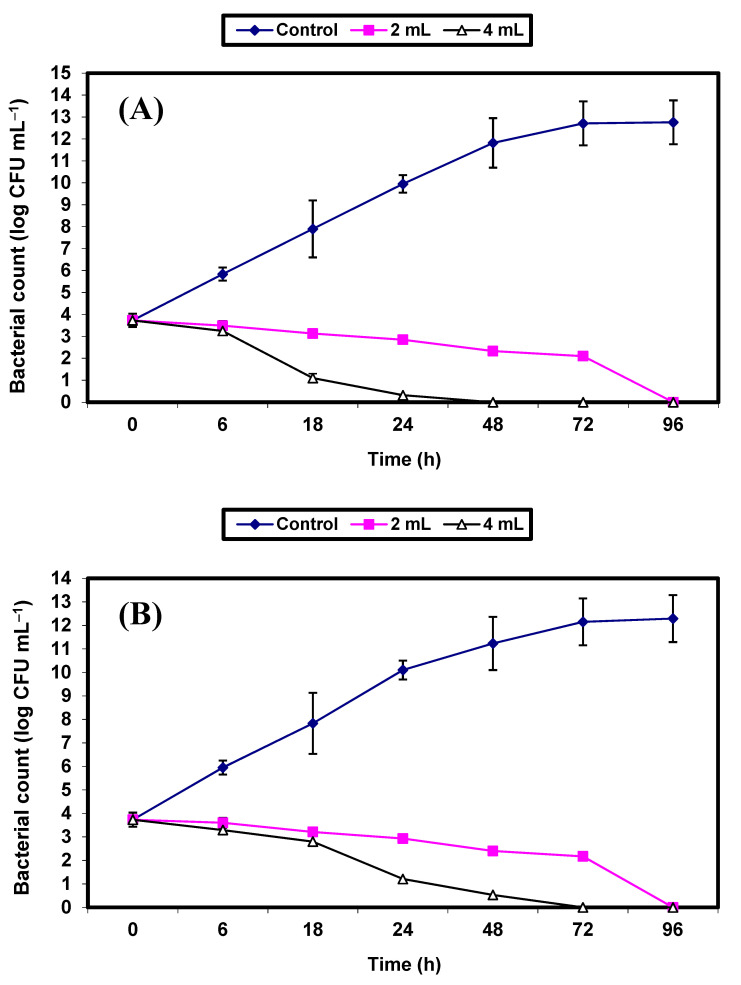
Inhibition of both *S. aureus* (**A**) and *E. coli* (**B**) in BHI broth. Symbols ♦; ■; ∆ refer to untreated control samples and juice samples treated with 2% and 4% KFB, respectively.

Since the *S. aureus* bacterium (a Gram-positive bacterium) and the *E. coli* (a Gram-negative bacterium) were the most sensitive bacterial strains to the antimicrobial potential of the KFB preparation among the kombucha preparations, they were nominated for further experiments to demonstrate the efficacy of KFB in controlling the growth of both strains in BHI broth and fruit juices (apple, guava, strawberry, and tomato). Results on the inhibition of both bacterial strains by KFB in BHI broths are presented in Figure 3A,B. Growth of the control cells of either *S. aureus* (Figure 3A) or *E. coli* (Figure 3B) increased vigorously, reaching an increase of almost 9 log cycles within 72 h. However, growth of the treated cells of both bacterial pathogens in BHI broths treated by either 2% or 4% *v*/*v* KFB decreased significantly (*p* ≤ 0.05), and differences between values of log CFU/mL of controls and treated samples were almost 10 log cycles after 48 and 72 h in all treatments. No growth of *S. aureus* and *E. coli* was detected after 48 h and 72 h of incubation, respectively, in BHI broths treated with 4% KFB. No growth of either of the bacterial pathogens was detected after 96 h of incubation in BHI broths treated with 2% KFB (Figure 3A,B).

**Figure 4:**
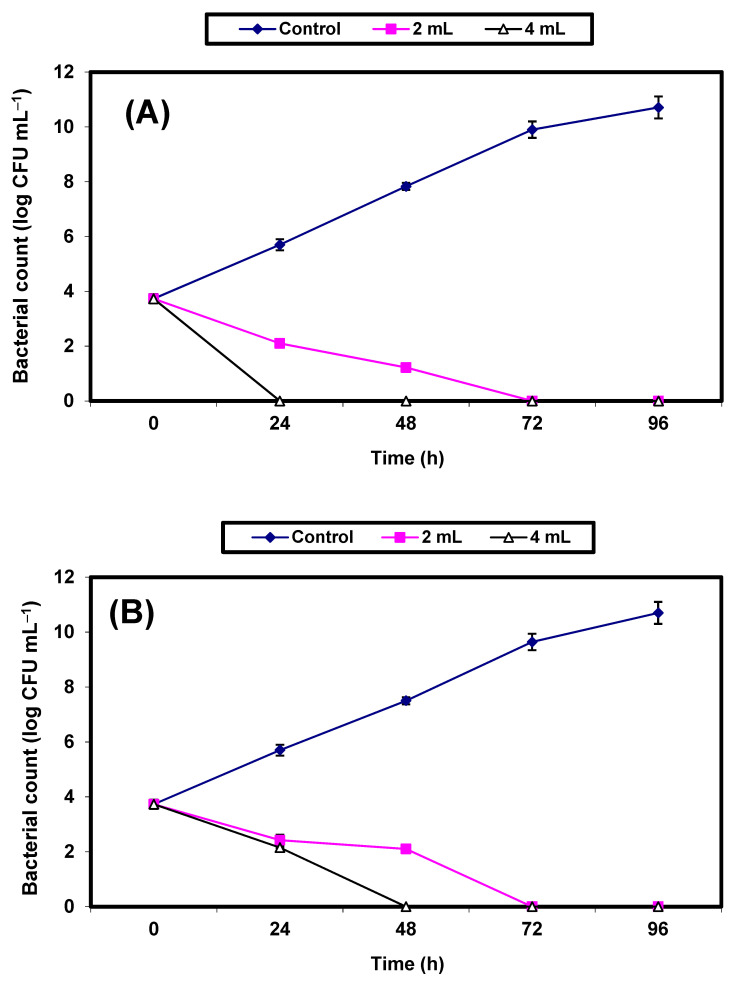
Inhibition of both *S. aureus* (**A**) and *E. coli* (**B**) in apple juice. Symbols ♦, ■, ∆ refer to untreated control samples and juice samples treated with 2% and 4% KFB, respectively.

In sterile apple juice treated with either 2% or 4% *v*/*v* KFB and inoculated with 7.3 × 103 CFU/mL of either *S. aureus* or *E. coli*, the growth of the control cells of both *S. aureus* (Figure 4A) or *E. coli* (Figure 4B) increased vigorously, with an almost 7 log cycles increase within 96 h. However, growth (CFU/mL) of the treated cells decreased significantly (*p* ≤ 0.05) and no growth was detected in either of the bacterial pathogens following 72 h of incubation in apple juice treated by 2% KFB. Treatment with 4% KFB showed no growth of *S. aureus* and *E. coli* after 24 h and 48 h, respectively (Figure 4A,B).

**Figure 5:**
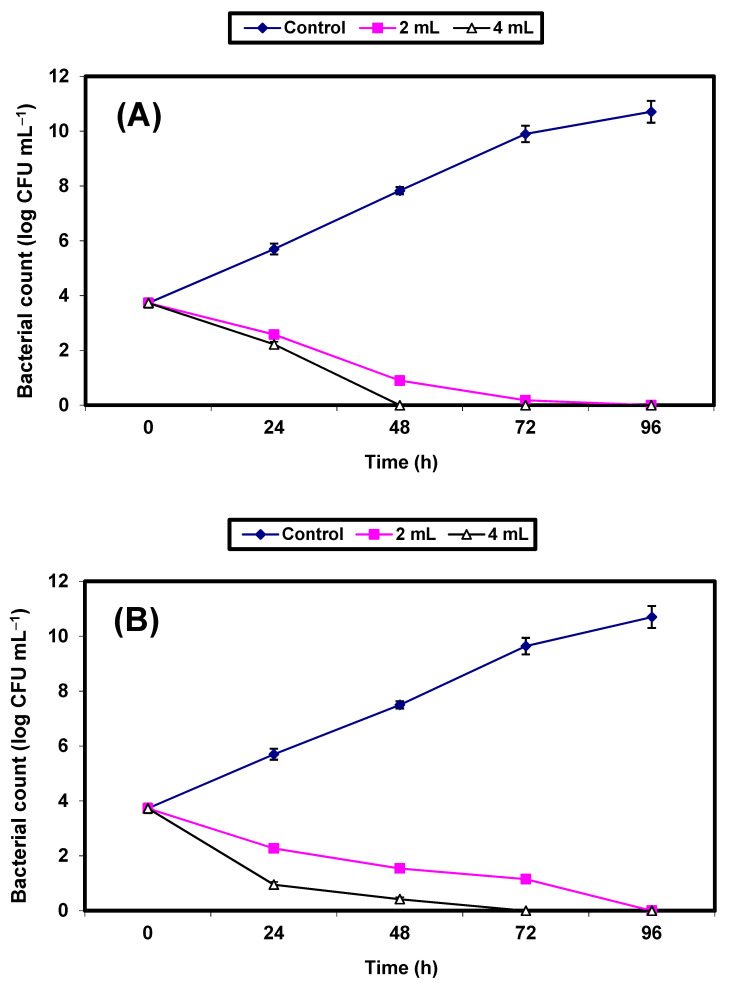
Inhibition of both *S. aureus* (**A**) and *E. coli* (**B**) in guava juice. Symbols ♦, ■, ∆ refer to untreated control samples and juice samples treated with 2% and 4% KFB, respectively.

The inhibitory effect of KFB (2% & 4%) on *S. aureus* and *E. coli* grown in fresh guava juice was investigated (Figure 5A,B). The control cells increased by almost 7 log cycles within 96 h, but growth (CFU/mL) of the treated cells decreased significantly (*p* ≤ 0.5), and no growth of either bacterial strain was recorded after 48 h and 72 h in guava juice samples treated with 4% KFB, or after 72 and 96 h in samples treated with 2% KFB, respectively (Figure 5A,B).

**Figure 6:**
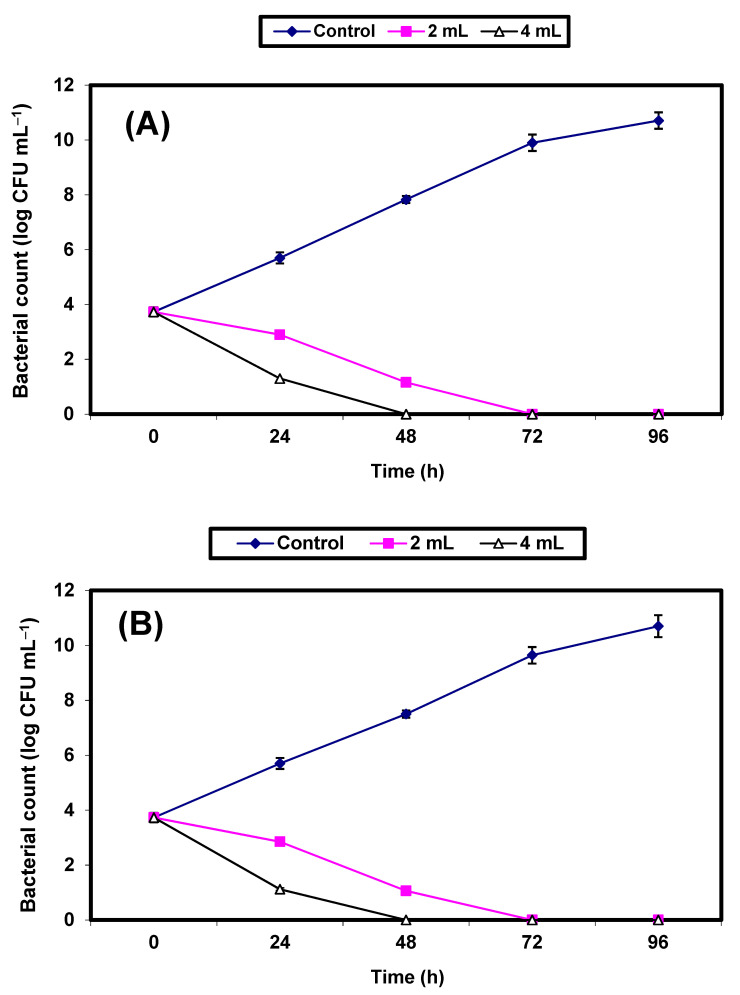
Inhibition of both *S. aureus* (**A**) and *E. coli* (**B**) in strawberry juice. Symbols ♦, ■, ∆ refer to untreated control samples and juice samples treated with 2% and 4% KFB, respectively.

Results of the effect of 2% and 4% (*v*/*v*) KFB on the growth of the two pathogenic bacteria strains inoculated in sterile strawberry juice are given in Figure 6A,B. The growth of the control cells increased, reaching almost 7 log cycles within 96 h for both organisms, but the growth of both pathogens decreased significantly (*p* ≤ 0.05), reaching zero after 48 h of incubation in samples treated with 4% KFB. In juice samples treated with 2% KFB, no growth of either strain was detected after 72 h of incubation (Figure 6A,B).

**Figure 7:**
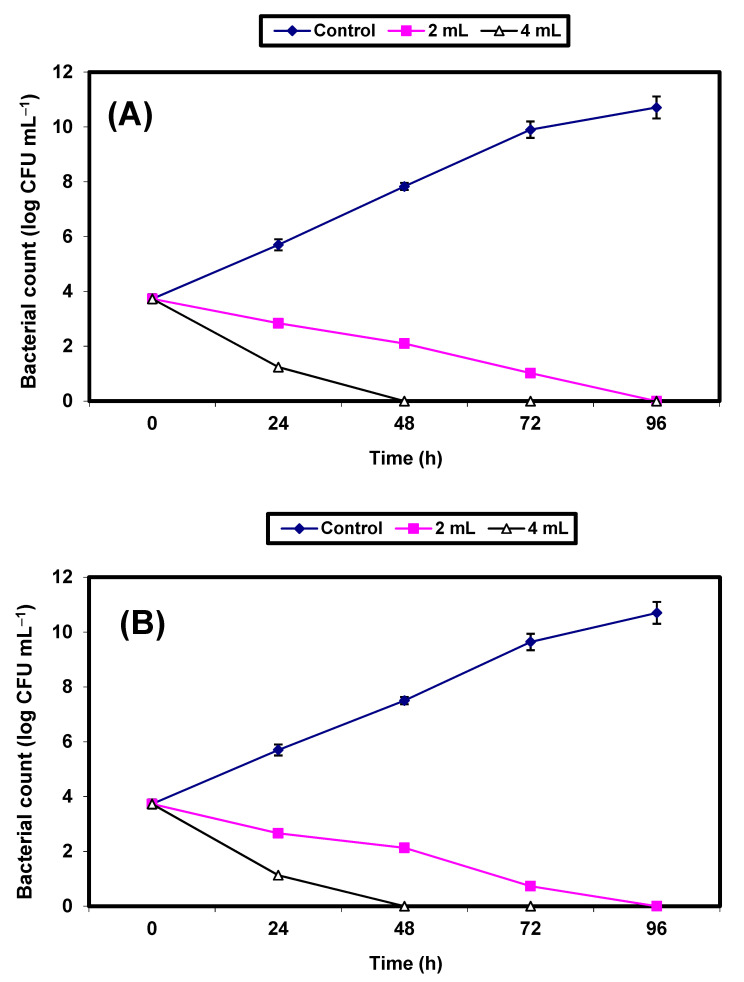
Inhibition of both *S. aureus* (**A**) and *E. coli* (**B**) in tomato juice. Symbols ♦, ■, ∆ refer to untreated control samples and juice samples treated with 2% and 4% KFB, respectively.

The inhibition of both *S. aureus* and *E. coli* in tomato juice by KFB (2% and 4%) was studied. Results are given in Figure 7A,B. The control cells increased by almost 7 log cycles within 96 h, but growth (CFU/mL) of the treated cells decreased distinctly (*p* ≤ 0.05), and no growth of *S. aureus* and *E. coli* was recorded after 48 h and 96 h, respectively, in juice treated with 4% and 2% KFB, respectively (Figure 7A,B).

## 3. Discussion

The kombucha used in this study was a traditional culture made by fermentation of black tea with kombucha mat (Egyptian made) [28]. Fermentation of kombucha results in the production of many fermentation end products. In this study, nine chemical groups were detected by GC-MS analysis; all of them were reported to inhibit bacterial pathogens by different mechanisms of action. However, it was found that the bioactive metabolites produced during the fermentation process of kombucha collectively have synergistic and cooperative effects [22].

Alkaloids have been reported to exert antibacterial activity by causing membrane damage and rapid denaturation of proteins, as well as nutrient leakage from the cell [29], thereby causing a defect in cell metabolism and cell lysis [30].

The esters and fatty acid esters detected herein were, in general, positively charged and more hydrophobic; such hydrophobicity allows electrostatic interactions with the bacterial cellular components, resulting in loss of cell viability via formation of fully deorganized killed cells. They also act as antibacterial food additives through the inhibition of bacterial growth and biofilm formation [10,31].

5-hydroxymethyl-2-furaldehyde (heterocyclic aldehyde) exhibited antibacterial activities against Gram-negative plant pathogenic bacteria; *Xanthomonas axonopodis*, *Pectobacterium carotovorum* subsp. *atrosepticum*, *Pectobacterium chrysanthemi*, *Erwinia amylovora*, and *Herbaspirillum rubrisubalbicans* [32]. The mechanism of antibacterial action of 5-nitro-2-furaldehyde (a furaldehyde derivative) was reported through its interaction with DNA secondary to formation of a nitro anion radical via one-electron reduction of the redox-active nitrofuran moiety [33]. Aromatic aldehydes detected in this study were reported to possess remarkable bactericidal activity through their binding with the outer layer of bacterial cells [34], specifically with unprotonated amines on the cell surface which in turn affect the transport of ions across the cell wall and on enzyme systems where access of substrate to an enzyme is prohibited [35].

Both unsaturated lactones and hydroxylactones detected herein KFB were found to exhibit antimicrobial activity against pathogenic strains of bacteria (*Staphylococcus aureus*, *Pseudomonas fluorescens* W1), yeasts (*Candida albicans* KL-1) and filamentous fungi (*Alternaria* sp., *Penicillium* sp.) [36]. The ability of lactone compounds to act as inhibitory substances against several microorganisms is due to their ability to penetrate the microbial cell and inactivate sulphydryl-containing enzymes necessary for cellular replication [37].

The antibacterial activity of heterocyclic compounds has been reported through their ability to interact with either electrophiles or nucleophiles of the cells, leading to the inhibition of cell wall synthesis, protein synthesis, DNA synthesis, metabolic pathways, and interference with cell membrane integrity [38].

The presence and quantity of the kombucha chemical constituents are variable, mainly depending on the microorganisms of the symbiotic culture used for fermentation of kombucha, as well as fermentation time and temperature, sucrose content and type of tea used, in addition to the analysis methods used for quantification [16,22].

In the current study, we isolated and identified bacterial isolates in the kombucha consortium. All bacterial identification was carried out automatically by Viteck-2 system (Vitek^®^ 2: Healthcare|Biomérieux, Marcy-l’Étoile, France) for the accuracy portion of the study. Three bacterial isolates of acetic acid bacteria from kombucha were identified on acetobacter agar medium which are *Acetobacter xylinum, A. pasteurians,* and *A. aceti*. Additionally, two lactic acid bacterial isolates were identified in this study; *Lactobacillus fermentum* and *L. acidophilus*. In agreement with our study, Mayser et al. [39] and Sievers et al. [40] reported the isolation of *A. xylinum* from the kombucha consortium. In another study, Marsh et al. [41] reported that the dominant bacteria in five kombucha samples from different origins were species of *Gluconacetobacter* and *Lactobacillus*. The most abundant bacterial species in this culture belonged to the bacterial genera *Acetobacter* and *Gluconobacter* [16]. In addition to bacterial isolates, two yeasts isolates, *Saccharomyces cerevisiae* and *Schizosaccharomyces pombe*, were identified by API-yeasts kits. The bacterial and yeast species encountered from kombucha in this study almost concur with previous published studies [16,22]. Similarly to the milk-derived kefir, the exact microbial composition of kombucha varies according to some factors such as the type of fermented substrate, the source of starter culture, the duration of fermentation process [10,16].

The data employed herein showed that the fermented preparation (KFB) from 14 days incubation of kombucha starter in black tea exerted the strongest antimicrobial activities. These activities were also exhibited after heating-treatment of kombucha beverage (HBK) but with a decrease in antibacterial and antifungal activity. The neutralized kombucha preparation (NKB) showed the lowest antimicrobial activities against the organisms tested. In support of the present study, Battikh et al. [23] demonstrated that fermented infusion of kombucha was superior in its antibacterial activity to unfermented, neutralized, acidified and heat-denatured infusion against pathogenic bacteria. They further showed that the heat-denatured preparation had more antibacterial activity against bacterial strains tested than the neutralized preparation. Several additional previous studies proved that kombucha of fermented black tea exerts antibacterial activity against a broad spectrum of bacteria [19,42,43,44]. Kaewkod et al. [45] found that the kombucha prepared from different types of tea (green, oolong and black) after 15 days of fermentation had efficient inhibitory activity on all pathogenic enteric bacteria tested: *E. coli* O157:H7 DMST 12743, *Shigella dysenteriae* DMST 1511, *Salmonella typhi* DMST 22842, and *Vibrio cholerae*.

A small number of studies in the literature have demonstrated the antifungal activity of kombucha. Sreeramulu et al. [20] reported that kombucha tea fermented for 6–14 days had antifungal activity against *C. albicans*. Battikh et al. [23] showed that fermented infusion of kombucha black tea for 21 days exhibited antifungal activity against *C. glabrata*, *C. tropicalis*, *C. dubliniensis*, and *C. albicans*. In a previous study, kombucha supernatant (10%, *v*/*v*) made from black tea after fermentation for 14 days caused a complete mycelium growth inhibition of *Acremonium implicatum* LC015097 and reduced the mycelial growth of *Penicillium expansum* LC015096, *Talaromyces purpureogenus* LC015095, in addition, it inhibited the production of the mycotoxin patulin by three fungal strains [28].

The antimicrobial activity exhibited by the KFB and HBK preparations may be explained on the basis of the acidity of organic acids (acetic, citric and gluconic acids) in kombucha beverage [17]. These organic acids, produced during fermentation from conversion of sucrose by yeasts and bacteria present in the kombucha consortium, shift the pH to final levels around 2.5–3.0, as recorded herein. This is strongly supported by previous studies confirming that increased concentration of organic acids produced in fermented kombucha broth made the pH decreased from 5 to 2.5 [20,46]. Moreover, Greenwalt et al. [42] showed that the antimicrobial activity of kombucha against pathogenic microorganisms is largely attributable to acetic acid. Organic acid molecules could induce cytoplasmic acidification and destroy bacterial cells [45]. The antibacterial activity exerted by kombucha compounds might also be interpreted on the basis of osmotic pressure of the solutes which existed in the hypertonic medium in relation to the outer aquatic medium; this facilitates the diffusion of the bioactive materials from cell membranes via the selective permeability. The lipophilic nature of some solutes facilitates their attachment to bacterial cell membranes which in turn causes cell death [47,48].

The lower antimicrobial potential of the NKB preparation compared to the KFB and HBK preparations implies that the inhibitory activity against bacterial and fungal strains was not due to acidic pH only, but also due to the other biological active compounds (proteins, antibiotics, alcohols, aldehydes, etc.) or metabolites other than acetic acid biosynthesized during the fermentation process of kombucha consortium. Such inhibitory biological compounds are heat sensitive, since the HKB preparation herein was tested as an alternative to KFB in order to characterize the nature of these compounds produced by kombucha. According to the present study, the inhibitory activity of kombucha infusions was not often due to heat-sensitive molecules; therefore, the antimicrobial activity of HKB preparation was either enhanced or reduced. This was clearly supported by Sreeramulu et al. [20] and Battikh et al. [23], and explained the weak antimicrobial activity exhibited by NKB preparation.

In all cases, *S. aureus* (a Gram-positive bacteria) and *E. coli* (a Gram-negative bacteria) were the most susceptible organisms for the antimicrobial activity of kombucha beverage preparations giving inhibition zones diameter of 19 and 18 mm, respectively in this study. Using agar diffusion assay, Battikh et al. [23] found that kombucha tea showed lower inhibition zone diameters of the two respective bacterial species (14.5 and 11 mm) than those obtained in this study. These data are very important, because both bacterial species are enteropathogenic microorganisms and are still the most prevalent and important public health problem in developing countries; in addition, they are the most common pathogens causing healthcare-associated infections and bacteremia and the treatment of their infections is becoming increasingly difficult due to emerging antimicrobial resistance [49,50,51,52,53,54].

In this study, *S. aureus* and *E. coli* were employed in further experiments concerned with the control of their contamination in BHI broth and juices such as apple juice, guava juice, strawberry juice, and tomato juice. Results obtained showed that KFB preparation significantly inhibited both bacterial strains in all trials. This is a promising result for the possibility of using KFB not only as a juice additive but also as a starter for obtaining protective syrup during vegetable or fruit juice fermentation. In a similar manner, Ayed et al. [55] developed a beverage from red grape juice fermented with the Kombucha consortium for 12 days that showed a remarkable antibacterial activity against *E. coli* ATCC 10536, *P. aeruginosa* ATCC 9027, *K. pneumoniae* ATCC 10031, *S. aureus* ATCC 6538, *S. epidermidis* ATCC 12228, *Enterococcus faecalis* ATCC 10541, and *B. cereus* ATCC 11778. The authors attributed the antibacterial activity was not due to polyphenolics, but rather to metabolites produced during fermentation such as acetic acid as well as other organic acids in KFB. In a previous study, KFB preparation was used efficiently for growth inhibition of *Acremonium implicatum* LC015097 (a strain causing decaying of apple fruits) and inhibition of mycotoxin patulin accumulation in liquid medium and apple fruits [28].

Finally, it is more advisable to use a safe probiotic drink like kombucha that contains several synergistic antimicrobial components than a single component that may be more expensive with a lower antimicrobial efficacy. This trend is promising and has applied feasibility within the functional food market. Therefore, GC-MS was used in this study as an analytical method that combines the features of gas-chromatography and mass spectrometry to identify different components within the KFB. Many volatile flavor compounds are produced during the fermentation of kombucha using black tea, and this is clearly supported by previous research [56], where 21 volatile flavor compounds were identified in the initial kombucha fermentation broth in raw tea and 56 volatile flavor compounds were identified after 10 days of fermentation [56]. Additionally, drying temperature, tea grade, and temperature–grade interaction showed a significant effect on the volatile composition of black tea [57].

## 4. Materials and Methods

### 4.1. Kombucha Consortium

The tea fungus starter culture used in this study, a traditionally Egyptian-made one, was a symbiotic culture between yeast and acetic acid bacteria producing a cellulosic pellicle layer floating on the surface of the fermented broth during the fermentation process of kombucha [28].

### 4.2. Preparation of Kombucha Fermented Beverage (KFB)

Kombucha fermented beverage (KFB) was prepared by adding 1.2% black tea (El Arosa Egyptian dust black tea) to boiling water in and left to infuse for about 5 min, thereafter the infusion was filtered through sterile sieve. Sucrose (10%) was dissolved in hot tea and the solution (200 mL) was left to cool, transferred into a sterile 500 mL glass vessel (22 × 20 × 15 cm). The preparation was inoculated with 3% (*w*/*v*) of freshly grown tea fungus that had been cultured in the same fermentation medium for 14 days and 10% (*v*/*v*) of previously fermented kombucha beverage asceptically. The glass vessel was carefully covered with a clean cloth and incubated at 30 °C for about 14 days in the dark to avoid oxidation of phenolic compounds [58]. The obtained kombucha beverage was filtered to remove the cellulose pellicle, and the supernatant was subjected immediately to both chemical and microbiological analysis. The obtained KFB aliquots used in the antimicrobial tests were stored in the refrigerator at 4 °C until used. After storage for a week, the KFB aliquots were surface-sterilized before applying the antimicrobial potency tests.

### 4.3. pH Determination

The pH of KFB was measured using an electronic pH meter (Denver Instruments, Bohemia, NY, USA).

### 4.4. Instrumental Analysis of KFB

The analysis of KFB existing chemical compounds was performed using Trace GC 1310-ISQ mass spectrometer (Thermo Scientific, Austin, TX, USA) with a direct capillary column TG-5 MS (30 m × 0.25 mm × 0.25 µm film thickness). The column oven temperature was initially held at 50 °C and then increased by 5 °C/min to 230 °C for 2 min and increased to the final temperature 290 °C at a rate of 30 °C/min and hold for 2 min. The injector and MS transfer line temperatures were kept at 250, 260 °C, respectively; helium was used as a carrier gas at a constant flow rate of 1 mL/min. The solvent delay was 3 min and the kombucha tea samples were filtered through a 0.22 µm sterile microfilter and 50 μL of the filtrate was injected automatically using Auto sampler AS 1300 coupled with GC in the split mode. The mass spectra were collected at 70 eV ionization voltages over the range of *m*/*z* 40–1000 in full scan mode. The ion source temperature was set at 200 °C. Compounds were identified by comparison of their retention times and mass spectra with those of WILEY 09 and the National Institute of Standards and Technology 2011 mass spectral library (NIST 11) [59,60].

Infrared spectra of the obtained KFB were measured with a Fourier transform infrared (FTIR) spectrometer (Bruker Optik GmbH, Ettlingen, Germany) according to the method reported by previous researches [61,62], to determine the presence of various functional groups in the obtained KB. The pellets for FTIR analysis were obtained by grinding a mixture of 1 mg of freeze-dried KB powder with 100 mg of dry potassium bromide powder (KBr), followed by pressing the mixture in a mold. The FT-IR spectra were recorded in the region of 4000–400 cm^−1^ at a resolution of 4 cm^−1^. The resulting data were processed using OPUS/IR NT4.0 spectroscopic software package (Bruker Optik GmbH) installed on the FTIR instrumentation.

### 4.5. Isolation and Identification of Bacterial Species from the Kombucha

Serial two-fold dilutions of the KFB were made; then 0.1 mL aliquots from these dilutions were pipetted onto specific *Acetobacter* agar (Oxoid, Basingstoke, UK); MRS agar plates [63,64]; Sabaraoud agar (Oxoid) for isolation of acetic acid bacteria; lactic acid bacteria; yeasts respectively. The agar plates were incubated at 35 °C for either 48 h for bacteria or 4 days for yeasts. Pure and single colonies of the obtained microbes were picked up by sterile needles and inoculated into Brain Heart Infusion broths (BHI broth, Oxoid). After 24 h of incubation at 35 °C, bacterial identification was carried out automatically by Vitek 2 system (Vitek^®^ 2: Healthcare|Biomérieux, France). The isolated bacterial species were checked for their Gram stain and cell morphology using a light microscope [65]. Yeasts were identified via morphological examination under light microscope and API-yeasts kits (BioMérieux, France) as given by the manufacturer’s instructions [66].

### 4.6. Microbial Test Strains

The microbial strains used in the antimicrobial tests included both bacterial and fungal pathogens. The bacterial strains used included Gram-positive bacteria such as *Staphylococcus aureus* ATCC6538 (*S. aureus*), *Bacillus cereus* ATCC14579 (*B. cereus*), *Listeria monocytogenes* ATCC4957 (*L. monocytogenes*), and Gram-negative bacteria such as *Escherichia coli* ATCC11229 (*E. coli*) and *Salmonella typhimurium* ATCC14028 (*Sal. typhimurium*). These bacterial test strains were maintained in glass beads, stored at −20 °C, and subcultured into brain heart infusion broth (BHI broth, Oxoid). The fungal test strains used, included *Aspergillus flavus* ATCC16872 (*A. flavus*) and *Aspergillus niger* ATCC20611 (*A. niger*). These fungal cultures were stored at −20 °C and subcultured onto potato dextrose broth (Difco, Sparks, NV, USA).

### 4.7. Antimicrobial Bioassays of Kombucha Beverage

The KFB was prepared as described above. It was also neutralized (NKB) at pH 7.0 by adjusting the pH with 1 M HCl or 1 M NaOH. Heat-denatured kombucha (HKB) was prepared by treatment of KFB at 120 °C for 15 min. The prepared kombucha samples were all centrifuged at 15,000 rpm for 15 min to remove cell debris. Sterile supernatants were obtained by filtering the supernatants through a sterile microfilter (Millex-GV filter, 0.22 µm pore size, Millipore, Burlington, MA, USA). The antimicrobial activities of all kombucha samples were studied using an agar well diffusion assay [67]. Brain Heart infusion agar plates (BHI agar, Oxoid) were prepared and inoculated by 7.3 × 10^3^ CFU/mL of the bacterial strains tested. Additionally, potato dextrose agar plates (PDA, Oxoid) were prepared and inoculated by 10^7^ spores/mL of the fungal strains tested. Microbial inocula were spread onto the agar plates by sterile glass rods under completely aseptic conditions. Wells of 10 mm diameter were made with a sterile cork borer. Sterile samples (100 µL) were then transferred into the wells of agar plates inoculated with tested strains. The plates were first stored at 4 °C for 2 h to allow a pre-diffusion of the kombucha preparation into the agar and treated agar plates were incubated at 35 °C for 48 h in the case of the bacterial strains and 5 days for fungal strains tested. Diameters of inhibition zones were then measured according to Clinical and Laboratory Standards Institute (CLSI) [68].

### 4.8. Preparation of Fruit Juices Employed in the Antimicrobial Activity of KFB

Fresh fruits of apple (*Malus domestica*), guava (*Psidium guajava*), strawberry (*Fragaria ananassa*) and tomato (*Solanum lycopersicum*) were purchased from local markets located in Zagazig City, Sharkia Governorate (80 Km north Cairo), Egypt.

Fresh fruits were washed with sterile distilled water. One hundred grams of each fruit sample were mixed with distilled water at the ratio of 1:1 (*w*/*v*) as described previously [69,70], then homogenized by using a mixer (Braun combimax 700 vital, Berlin, Germany). The obtained fresh juice was then centrifuged at 15,000 rpm for 30 min at room temperature. The supernatant of each fresh juice was collected in glass bottles, sterilized by autoclaving at 15 °C for 15 min and was then cooled and stored in refrigerator at 4 °C until used.

### 4.9. Inhibition of Both E. coli and S. aureus in BHI Broth and Juices of Apple, Guava, Strawberry and Tomato

A series of 250-mL Erlenmeyer flasks; each containing 100 mL aliquots of either BHI broth (Oxoid) or fruit juices were sterilized by autoclaving at 120 °C for 15 min. After cooling, they were separately inoculated with 7.3 × 10^3^ CFU/mL of either *E. coli* or *S. aureus*, treated by either 2% or 4% KFB, and were then incubated in an incubator (New Brunswick Scien. Co., North Brunswick, NJ, USA) at 30 °C for 4 days. Every 24 h, samples were withdrawn and the growth of the indicator bacteria (CFU/mL) was calculated as described previously [67].

### 4.10. Statistical Analysis

Results were expressed as the mean ± standard deviation (SD). Statistical significance was evaluated using analysis of variance (ANOVA) test (SAS version 9.1, SAS Institute, Inc., Cary, NC, USA) [71] followed by the least significant difference (LSD) test at 0.05 level (*p* < 0.05 means significant) [72].

## 5. Conclusions

In this study, the KFB was chemically analyzed by GC-MS and IR spectra, which was shown to contain nine chemical groups. These metabolites produced during the fermentation process collectively appeared to be responsible for the antimicrobial activity of kombucha in a synergistic action. KFB preparation exhibited a remarkable antimicrobial activity against some pathogenic bacteria and fungal strains using agar well diffusion technique and it was superior in its activity when compared with NKB and HKB preparation. This study offered new insights on application of KFB as safe alternative biopreservative for protection against pathogenic bacteria in fruit juices.

## Figures and Tables

**Table 1:** Putative identification of the chemical components from KFB when subjected to GC-MS (gas liquid chromatographic–mass spectrometry).

	Classification and Compound Name	Mol.wt & Mol. Formula	Parent Ion (M^+^)	Area	Base Peak (*m*/*z*) (100%)
	**Group A: Heterocyclic Alcohols**				
**1.**	2-Hydroxy methyl furan	C_5_H_6_O_2_ (98)	99	1.35	98.0
**2.**	2-(4-Hydroxyphenyl) ethanol	C_8_H_10_O_2_ (138.0)	138.0	0.83	1.07
	**Group B: Heterocyclic Acids**				
**1.**	1,2,3,4-Tetrahydro-2,4-dioxo-5-carboxy methyl uracil	C_6_H_6_N_2_O_4_ (170.0)	170.0	0.30	112.0
**2.**	Hexadecanoic acid CH_3_(CH_2_)_14_-COOH	C_16_H_32_O_2_ (256.0)	256.0	0.66	73.0
**3.**	Ethyl-2-[2,2-dimethylcyclopropanecarboxylate (cyclopropane carboxylic acid)	C_10_H_14_Cl_2_O_2_ (236.0)	236.0	0.42	163.0
	**Group C: Lactone**				
**1.**	3,5-Dihydroxy-6-methyl-2,3-diydroxy-4H-pyran-4-one	C_6_H_8_O_4_ (144.0)	144.0	0.85	43.0
**2.**	4-Hexyl-2,5-dihydro-2,5-dioxo-3-furan acetic acid	C_12_H_16_O_5_ (240.0)	240.0	0.12	126.0
**3.**	3-Methoxy-2,4,6-trimethylcyclohex-2-enone	C_10_H_16_O_2_ (168.0)	168.0	0.12	126.0
**4.**	3,7-Dimethyl-1-[2-(vinyloxy) ethyl-3,7-dihydro-1*H*-purine-2,6-dione	C_11_H_14_N_4_O (250.0)	250.0	0.55	180.0
**5.**	S-[(2E)-1,3-diphenyl-2-butenyl] dimethylthiocarbamate	C_19_H_21_NOS (311.0)	311.0	0.22	207.0
	**Group D: Condensed Heterocyclic cpd**				
**1.**	2,3-Dihydrobenzofuran	C_8_H_8_O (120.0)	120.0	0.47	0.91
**2.**	7,7-Dimethyl-1-Isobutyl hexahydro-2-benzofuran-3a (3*H*)-ol	C_14_H_26_O_2_ (226.0)	226.0	0.51	159.0
	**Group E: Antibiotics**				
**1.**	Cypermethrin	C_22_H_19_Cl_2_NO_3_ (415.0)	415.0	0.30	163.0
**2.**	Cyhalothrin	C_23_H_19_Co F_3_NO_3_ (449.0)	449.0	0.30	181.0
	**Group F: Heterocyclic Esters**				
**1.**	5-Acetoxymethyl-2-furaldehyde	C_8_H_8_O_4_ (168.0)	168.0	0.38	126.0
	**Group G: Heterocyclic Aldehydes**				
**1.**	5-Hydroxymethyl furfural	C_6_H_8_O_3_ (126.0)	126.0	13.45	97.0
	**Group H: Unsat. Fatty Acids**				
**1.**	2-Hexadecenoic acid	C_16_ H_30_O_2_ (254.0)	254.0	0.40	43.0
	**Group I: Alkaloids**				
**1.**	1,3,7-Trimethyl-3,7-dihydro-1*H*-purine-2,6-dione (caffeine)	C_8_H_10_N_4_O_2_ (194.0)	194.0	69.24	109.0

**Table 2:** Antimicrobial activity of unfermented, fermented, neutralized and heat-denatured kombucha determined by the agar well diffusion method.

Tested Organism	Inhibition Zone Diameters (mm)				
	UKB	KFB	NKB	HKB	*p*-Value
*Salmonella typhimurium* ATCC14028	N.A.	14.0 ± 0.2	1.3 ± 0.2	10 ± 0.1	00.000
*List. Monocytogenes* ATCC4957	N.A.	15 ± 0.0	1.0 ± 0.0	7 ± 0.3	0.000
*B. cereus* ATCC14579	N.A.	14.5 ± 0.18	1.4 ± 0.0	8.5 ± 0.0	0.000
*S. aureus* ATCC6538	N.A.	19 ± 0.1	7 ± 0.0	12 ± 0.1	0.000
*E. coli* ATCC 11229	N.A.	18 ± 0.25	5.3 ± 0.0	12 ± 0.0	0.000
*A. flavus* ATCC16872	N.A.	12 ± 0.0	1.1 ± 0.1	5.0 ± 0.0	0.000
*A. niger* ATCC20611	N.A.	9.5 ± 0.0	0.9 ± 0.2	4.6 ± 0.0	0.000

ATCC: American Type Culture Collection. N.A.: No activity.

## Data Availability

The data presented in this study are available in the article.

## Sample Availability

Samples are available from the authors.
